# Adolescent pregnancy amongst displaced women in Bogota: playing between the barbs of structural violence—a qualitative study

**DOI:** 10.1186/s12978-023-01731-8

**Published:** 2024-08-13

**Authors:** Nicola Didi Wallis, Yazmin Cadena Camargo, Anja Krumeich

**Affiliations:** 1 Department of Health, Ethics and Society, Research School CAPHRI, Faculty of Health, Medicine and Life Sciences, Maastricht University, Maastricht, The Netherlands; 2 Department of Preventive and Social Medicine, Faculty of Medicine, Pontificia Universidad Javeriana, Bogotá, Colombia

**Keywords:** Adolescence, Pregnancy, Structural violence, Conflict, Internal displacement

## Abstract

**Background:**

Colombia has high numbers of internally displaced people, forced to migrate due to the conflict. 1 in 3 displaced women undergo pregnancy during adolescence, compared to around 1 in 5 in the non-displaced population, alongside health and resource inequalities between these groups. There is limited qualitative information available from the perspectives of displaced women experiencing adolescent pregnancy. This research explores how structural violence may feature in their experiences.

**Methods:**

Qualitative methods were used. Participants were recruited with purposive sampling, using key informants and snowball sampling technique. 14 semi-structured interviews were conducted in Ciudad Bolívar, Bogotá, involving 11 displaced women who began childbearing age 15–19 in the past 10 years, and 4 participants’ mothers. Data was analysed using the theoretical framework of structural violence, and emergent themes categorised using thematic analysis.

**Results:**

Pregnancy was considered advantageous in many ways, but this was contradicted by resulting disadvantages that ensued. Structural violence was embedded in life stories, manifesting in poverty and difficulties accessing reliable income, poor access to healthcare and education following pregnancy. Institutional and interpersonal discrimination confounded these challenges.

**Conclusions:**

Pregnancy during adolescence was a contradictory experience, representing both a safety net and a trap due to a complex interplay of structural and cultural violence in everyday survival. Policymakers must consider the importance of the context surrounding adolescent pregnancy and address systematic disadvantages affecting women in these positions.

## Plain language summary

The violent conflict in Colombia has left many people forced to leave their homes and become ‘internally displaced’. Internally displaced women are more likely to become pregnant during their adolescence than non-displaced women. This work tries to understand more about the everyday lives of displaced women who experience adolescent pregnancy, through interviews. The interviews were analysed and results interpreted using the theory of ‘structural violence’. Structural violence describes how social structures such as racism, sexism, war and poverty determine life choices, leading to suffering and inequality. The work found that pregnancy and motherhood in adolescence for displaced women was positive in many ways by bringing purpose, status and companionship. However, these women also experienced many challenges after pregnancy, such as exclusion from education and secure employment and difficulty accessing healthcare. This demonstrated that structural violence features in multiple interconnected forms in the daily lives of displaced adolescent mothers. The work urges policymakers to appreciate the complexity of context surrounding adolescent pregnancy and motherhood, and to address the structural disadvantages facing women in these situations.

## Background

Colombia is considered to have high rates of pregnancy among adolescents [1, 2]. The Colombian demographic and health survey, *Encuesta Nacional de Demografía y Salud* [ENDS], published data in 2015 stating that 17.5% of 15–20 year-old Colombian women had started child bearing [3]. The highest rate of adolescent pregnancy is among the internally displaced population, the 8.5 million people who have been forced to leave their homes as a result of the decades of violent conflict [4]. According to data from 2005, up to 1 in 3 displaced women between 15 and 19 had started childbearing, compared to 1 in 5 from the general population at that time [5, 6]. More recent research has demonstrated that displacement was directly associated with an increased risk of adolescent pregnancy compared to the non-displaced population [7].

Pregnancy during adolescence has been represented as problematic in health and social contexts globally, leading to adolescent pregnancy being described as ‘a violation of human rights’ by the United Nations Population Fund [UNPF] [8]. This negative representation is based on an association with certain health, social and economic outcomes. Pregnancy during adolescence is associated with a higher rate of complications during pregnancy and birth, with birth complications representing a significant cause of mortality amongst 15–19-year-old women worldwide [9–12]. Many of these complications can be related to social and economic factors such as nutritional status and non-attendance to adequate antenatal care, with inadequate antenatal care strongly associated with worse pregnancy outcomes [12, 13].

Adolescent pregnancy is also represented as problematic due to an association with particular social and economic outcomes. Women across Latin America who have children during teenage years are 3 times less likely to acquire university level education and on average earn 24% less than those who start childbearing over the age of 20 [2]. This association does not infer causation however, and rates of teenage pregnancy in Colombia are higher among women in the lower quintiles of wealth and in those in rural settings [3], making it difficult to establish whether these outcomes are related to adolescent pregnancy itself or other social disadvantage before and after pregnancy [14]. The representation of adolescent pregnancy as a problem has been challenged in the field of social science, with the idea that the social circumstances surrounding pregnancy are more important, rather then adolescent age alone being the problem [15].

Both national media and international organisations describe the figures in Colombia as ‘alarming’, and adolescent pregnancy and motherhood are frequently represented as negative phenomena in the Colombian media [2, 16, 17]. This narrative has been previously posited as promoting negative views on adolescent pregnancy as a means of population control of poorer Colombian women to improve national economic growth [16]. This situation prompts the question as to whether the age at which women become pregnant is, in itself, problematic, or whether particular social contexts or other structural forces contribute to adolescent pregnancy becoming problematic.

National and international policies, such as the Montevideo Consensus, have focused on reducing the rate of adolescent pregnancy, with a specific focus on prevention through sexual and reproductive education, and improved access to contraception for adolescent women [8, 18–20]. Intensified public health initiatives in Colombia have been deemed successful in recording a fall in the rate of adolescent pregnancy among 15–19-year-olds from 20.5% in 2005, to 17.4% in 2015 [3]. However, previous qualitative studies in the displaced population have demonstrated that the routes to pregnancy during adolescence were more complex than just the lack of access to contraception or sexual health services and demonstrated agency in reproductive choices in this group [21, 22].

This complexity makes it necessary to understand the context of life for displaced adolescent mothers, as a specifically vulnerable group in Colombia, when forming policy. Despite the rate of displaced women experiencing adolescent pregnancy and motherhood, there is limited qualitative information available from their perspectives. This research aims to generate a deeper understanding of the lives of displaced women experiencing adolescent pregnancy, identify ways in which it may or may not be problematic for them and what the focus of policy and intervention should be. The lens of structural violence has proven fruitful in exploring connections between social and health disparities and we will be using this as a theoretical framework [23].

### Conflict and displacement in Colombia

Since 1985, the violent conflict in Colombia has resulted in 8.4 million people becoming internally displaced, corresponding to 16% of the population [24]. Over its 60-year course, the conflict has involved a multitude of actors with various motives, including state security forces and paramilitary groups, as well as guerrilla forces, political and corporate elites and drug traffickers [25–27]. In areas where armed groups are fighting for territorial control, local populations have been victims of killings, rape, torture, kidnappings and forced recruitment to armed groups [25, 28]. These activities deliberately terrorise civilians to force them to evacuate the area, enabling armed groups to extend territory and gain financial, social and political control of the land [25, 29]. Both Colombian and international large-scale infrastructure developments and extractive industries have made gains from areas with high amounts of forced displacement through paramilitary group activities, in areas with profitable natural resources [25, 30].

The peak of forced displacement was seen between 2000 and 2004, and started to decline as the government began negotiations to disband certain paramilitary groups in 2006 [24]. In 2012 the Colombian government began negotiations with the largest guerrilla group, the Revolutionary Armed Forces of Colombia (FARC), and in 2016 a historical peace agreement was signed between the FARC and the Colombian government [31]. Despite an initial reduction in violence in the years following the peace accord, levels began to escalate once more, and rates of violence and forced displacement in 2022 were similar to those prior to 2016 [24, 32].

Displacement predominantly occurs from rural areas to cities, with more than half of displaced civilians relocating to informal settlements in urban centres [3, 33]. The majority of displaced people move to informal housing with low access to resources, healthcare and employment [33, 34]. Here, violence and danger persist as a result of intra-urban conflict for territory among gangs [34]. The displaced population often struggle in unregulated labour markets and find difficulty accessing the over-subscribed governmental poverty relief programs [35]. Statistics from 2015 showed 92% of the displaced population lived below the poverty line, with 33% in extreme poverty, compared to 6.2% of the population generally [34].

The Colombian government provides financial support to victims of the conflict, enacted through the 2012 Victims Law (Law 1448), alongside reparations in the form of compensation for losses and restitution of land and property [36, 37]. Displaced citizens must first register their displacement, and once registered are eligible to receive emergency financial assistance for the first year after displacement, followed by transition assistance to those who have ongoing housing and subsistence needs thereafter [38]. Research in 2022 showed that only around 65% of the internally displaced population were receiving any form of governmental financial assistance, and over half of these were not receiving any financial support prior to the new scheme introduced after the 2020 pandemic [38]. This demonstrates how the poverty relief programs available to the displaced populations have been slow to reach all those requiring it.

The conflict predominantly affects the rural peasant or ‘campesino’ population, Afro-Colombian and indigenous communities [3, 25]. It has disproportionately affected women, due to the existence of gender-based violence and widespread sexual violence against women, as well as women being undervalued economically in an unequal system [31, 32]. Studies in Antioquia, an area with a high level of displacement, have shown that 41% of women had been exposed to physical violence, 39% to underpay, 13% to sexual violence and that 31% of pregnancies were unwanted [31]. There have been calls to address these issues through improvement of sexual and reproductive health education and access to sexual and reproductive health services, but also with elimination of gender stereotypes and support of women’s community-based organisations to tackle these problems [31].

### Structural and cultural violence

Structural violence is a theoretical framework used to understand potential mechanisms of inequalities among populations, initially described by Galtung in 1969 [23, 39, 40]. The theory explains how social structures such as racism, sexism, political violence, war and poverty constrain individuals and determine life choices, leading to avoidable suffering and resulting in an uneven distribution of resources and power [39]. ‘Resources’ may include financial resources, healthcare services and education. Uneven distribution of resources leads to skewed rates of morbidity and mortality amongst the population [23]. This theoretical framework was expanded in 1990 to include “cultural violence” to articulate how culture (for instance via cultural beliefs regarding gender roles) can serve to justify and legitimise existence of direct or structural violence [40].

In Colombia, disparities between the displaced population and the general population are well-documented. Alongside higher rates of adolescent pregnancy the displaced population faces disproportionate rates of health problems [41]. Moreover, the displaced community experience discrimination and stigmatisation in Colombia due to association with the conflict, amongst other tensions with recipient communities [33, 35]. In addition, pregnant adolescents are known to be victims of discrimination elsewhere [14, 42]. While we see different outcomes in terms of education and incomes between women who become pregnant in adolescence and those who do not, it is important to understand how structural and cultural violence, in the forms of discrimination and prejudice, may affect adolescent mothers in life prior to displacement and afterwards. Using the theoretical frameworks described by Galtung, this research aims to explore how structural and cultural violence may feature in the lives of displaced women experiencing adolescent pregnancy and motherhood.

## Methods

To gather insight about the context of the experiences of participants, qualitative methods were most appropriate [43]. We used tools based on ethnographic methodology to gather information from the perspective of the cultural group involved.

Participants included women who had started childbearing between 15 and 19, (as per the WHO factsheet [9]), currently in the situation of displacement. For the data collected to reflect current circumstances, the study included women who had experienced adolescent pregnancy and motherhood within the preceding 10 years. To gain a cross-generational perspective, mothers of the primary participants were also invited for interview.

The city of Bogotá has received the highest number of displaced people in the country [44]. The research was carried out in Ciudad Bolívar, a locality in south Bogotá, which is home to the most displaced persons within the city [45]. Ciudad Bolívar has undergone rapid growth in recent decades, due to spontaneous urbanization arising from the influx of large numbers of displaced persons [45]. The area has a high population density with large numbers of informal settlements, poor urban planning and there is low access to services[45]. According to government data, within this locality 53.1% of the population fall into the lowest economic classification, there is the highest proportion of people whose basic needs are not met (16.2%) and the access to health services is reported to be the lowest in the city [46].

Sampling was carried out through purposive and snowball sampling [47]. Participants who satisfied the inclusion criteria were identified by key informants from a community centre in Ciudad Bolívar. The women identified were invited to participate in the study with an interview and, where possible, their mothers were invited to participate [47]. Snowball sampling was also used, where known participants were asked to invite contacts who fulfilled the inclusion criteria to participate. The resultant population was sufficiently heterogenous in their experiences and backgrounds, with participants having been displaced from a variety of different regions. Sample size was determined through saturation[48].

Data was collected through in-depth interviews, allowing for information regarding specific aspects of structural violence to be discussed and also for other themes to emerge [43]. 14 interviews were carried out, between June and August 2016, involving 11 displaced women who had experienced adolescent pregnancy within the last 10 years, and 4 of the participants’ mothers.

Participants were asked about their daily life, the neighbourhood where they lived, employment, education, healthcare and experiences of pregnancy. They were also questioned about relationships with partners, their displacement, their perceptions of adolescent motherhood and asked to identify any problems that they faced in their lives. All interviews were recorded and were conducted by the first author, with the second author or a Colombian interpreter present to facilitate translation. All interview recordings were transcribed into English, and the transcripts were analysed. Data analysis was carried out using a combination of deductive and inductive codification. Deductive codes were identified prior to analysis using those described in the theoretical framework of structural and cultural violence by Galtung (1969). Inductive codes were also identified from data, allowing for emergence of new themes. Codes were categorised and categories were grouped into themes, using thematic analysis [49]. The emergent categories were discussed and analysed with the other authors to triangulate the data.

This research was approved by the ethics committee of the School of Medicine of Pontificia Universidad Javariana, in Bogotá D. C. (Act. 21/2015. FM-CIE-8744-15). All participants were briefed to ensure that they were fully informed of what the research entailed and how the information gathered would be used. Participants were provided with written information about the research, and the contact details of the researcher. Written consent was obtained from all participants. For any participants who were younger than 18, consent was also obtained from a parent as a representative. All names have been anonymised to maintain confidentiality.

## Results

We will present three main themes which emerged in the interviews. Firstly, there are the early experiences of adversity, with pregnancy as a perceived way out. Secondly, there is the of stigmatisation faced as a displaced adolescent mother and the challenges due to cultural and social systems. Thirdly, there are the barriers to economic self-sufficiency encountered by displaced adolescent mothers. After presenting these main themes, we will discuss and interpret the results through the framework of structural violence and explore how this relates to the problematisation of adolescent pregnancy.

### Early experiences of adversity: poverty, abuse and partnerships with older men; pregnancy as a way out

Participants had come from rural backgrounds. Some participants had begun childbearing whilst still living in rural areas, whereas others had had their first pregnancy after displacement.

The women described growing up in resource-poor environments, recounting experiences of poverty from early life. Although food was plentiful due to the access to space and natural resources, limited household income constrained existence, contributing to early experiences of suffering.

Abuse from family members during childhood and adolescence emerged as a common difficulty. As a result of abuse, low resources, or both of these, leaving home during childhood was an option to escape the difficulties of home life.

*‘I left my house when I was small, when I was only 8. My stepfather was very aggressive with us, with my mum as well and he abused us a lot […] He would take us out on the farm and force us [to work] hard, a lot of times he would arrive at the farm drunk, he hit us, he hit my mum, it was like that… And so because of that I had to go, little girl left home.’ Rosa, 27, first child age 19*

Poor access to resources rurally, familial household poverty and abuse restricted life choices, from early life. Faced with these challenges, women had to seek alternative forms of support as a means to survive and to improve the circumstances in which they existed. Seeking a relationship with an older male partner represented an opportunity to escape difficult circumstances within an abusive or impoverished household. A partner was seen as a provider of financial and emotional support. Amongst the women interviewed, first partnerships were universally with men who were older than them. Older partners represented greater potential to provide financial support, because of their age and social status.

Some women felt they had had inadequate knowledge or access to sexual and reproductive health services and so pregnancy may have been unexpected and unplanned. However, many narratives involved autonomous decisions rather than simply difficulty accessing contraception. In some cases, a pregnancy was a means to solidify a relationship and secure financial support from a male partner. Amongst other decisions, male partners could have power over the decision to have a child, some directly asking for pregnancy. Some women described going ahead with the pregnancy in order to secure support from the partner thereafter.

*‘After 2 years being with him he said that he wanted to have a baby, how nice it is [...] so – I had that problem. So, after 2 years being with him, [...] I said to him “ok if you’re going to look after me…” so I had a baby with him.’ Veronica, 23, first child age 16*

Whether pregnancy was intended or not, many of the participants felt that it was more acceptable for them, or more ‘normal’ to start childbearing during adolescence when they were living in the countryside, prior to displacement.

*‘When I was [in my pueblo (rural village)], there were people who were pregnant at 11 years old. And I fell pregnant, after I left home, after some time of travelling so, I don’t know if its so unusual to want to be pregnant.’—Yessica, 21, who had her first child at 15*

*‘Young motherhood was normal there. Normal. Because over there they already [raise children], since their childhood, working. In the countryside a child, at 6 or 7 already has to be grown up […] you raise children as your job. So that’s already your route, your path.’—Mariana, 36—mother of participant*

The sense that pregnancy was more acceptable in rural situations was influenced by the implications of their pregnancy and the perceived gained or lost opportunities that motherhood brought about. Women’s expectations from life rurally were relatively prescribed—motherhood was considered inevitable and work would involve domestic labour in the home and on one’s own land. Rurally, attending school was less available and considered less important. Thus, the age at which women started child-bearing had few implications for their ability to survive day-to-day, since there was always sufficient food and the ability to cultivate one’s own crops.

The experience of pregnancy was described as something positive and ‘beautiful’, irrespective of age.

*‘When they told me I was pregnant it made me happy – very happy! In spite of [the fact that] being pregnant [meant] I would have to make a lot of sacrifices. But, […] each little kick the baby was giving me gave me joy, every time that I had a scan I cried, from pure emotion. And well, it was a really beautiful experience’ Yessica, 21*

Amongst the benefits was many women’s feeling that the responsibility of motherhood brought them a new sense of purpose, and represented a change in social status, from that of the child into a mother, signifying entry into adulthood because of the new responsibility.

*‘I already want to have my children [...] I want to have this responsibility of my own. And [know] how it feels to have this devotion.’ Juliana, 19 who had recently had her first child.*

Completing the challenges of a perceived inevitable motherhood early in life was felt to be positive, so having children comparatively early meant that mothers were still youthful when their children were growing up. This was felt to allow a different kind of relationship with children to that of an older mother and children formed a valuable support system.

*‘[When I found out about the pregnancy], well, it was something exciting. Exciting because I wanted to have someone who was my company. Someone to be my company who would look after me, and all that.’ Yessica, 21.*

Just as the quote from Yessica shows, having children brought companionship. By providing purpose, dependence and devotion, motherhood remained a solution to surviving the difficult circumstances facing these women in rural life and subsequent displacement.

### The challenges of pregnancy: stigmatisation and the cultural construction of virtue

Despite the sense that adolescent pregnancy was presented as natural, inevitable and beneficial, women also discussed its challenges. Pregnancy was met by gossip and judgement from the rural communities.

*“They judge people there, they judge a lot. And everyone talks a lot, if someone falls pregnant. [...] [They judge people] primarily because of being a single mother. Because they say, ay look she has a child, and where is the father? […] they were just very critical there, [...] to be pregnant young and single, without a husband without anything, everyone talked about you, everyone criticises you.” Veronica, 23*

As this quote demonstrates, age was not the primary reason for gossip, which more predominantly stemmed from being a single mother, out of marriage or union and, as a single mother, being perceived as being irresponsible or incapable. This response represents a cultural belief in the nuclear family structure as the accepted form of a social unit. Engaging in this social norm reduced the scandal and community discrimination that can result from a ‘deviation’ from this family structure. For those who were still living with their parents, in rural settings or post-displacement, this often meant that they would be forced to leave their parents' household on discovery of a pregnancy.

*“[When you find out you are pregnant] you already know what is going to happen to you - that you will be kicked out the house. Because my mum said to me if you are pregnant you have to leave the house immediately so I was scared about that. My granddad was the same with her […] [When I found that] I was pregnant, [my mother] went to talk to my step-father, and he said that I had to leave home, to live with him [the baby’s father]” Natalia, 15—currently pregnant with her first child*

The belief system which upholds this practice could legitimise the suffering of younger women who become pregnant by forcing them to leave their parent’s house often against their will. The sudden upheaval from family life to a new partnership and a new role shifted the social position from being a child or daughter to being an adult or mother. Once in the social position of mother, continuing with school became less socially acceptable.

Although partnerships with older men offered both a route to escape familial poverty or abuse and brought the chance to avoid stigma and exclusion following pregnancy, women also reported difficulties in their relationships. Male-partners controlled decision-making regarding school and finances, as well as influencing women’s reproductive choices. The power imbalance potentially leads to mistreatment and abuse, both physical and financial, from partners, which emerged in the experiences of the women interviewed.

*‘When I got pregnant, I told the father of my children that I was pregnant, and he helped me throughout the pregnancy. Since I was a minor - you see he was much older than me, he was 36 years old. He helped and everything. But how do I say… he kept trying to control me… he hit me once. He hit me and left me with a black eye. Imagine! […] So I left him. I separated from him. I mean immediately after I had the baby, that was it, I separated from him.’—Camila, 34, mother of participant*

*'He didn’t take care of me, he didn’t let me eat and it wasn’t important to him if I ate or not, [even though I was] pregnant. […] He hit me twice. When we were living together. And so I was frightened and I didn’t tell anyone. I didn’t tell anyone.’—Nathalia 15*

There was also subordination of women within their home life.

*‘He was almost never responsible. I would say to him to buy things or clothes for the baby, and he almost never bought anything for her, and he would say no what is the baby going to do with this?’ Ana, 25*

Ana’s quote shows male control of financial resources and unequal power in decisions regarding running the household, as well as physical abuse reported. Stories of abuse from partners arose often as a reason for the end of relationships. All but two of the women were no longer in a relationship with the father of their first child, and subsequently women faced the stigma of being a single mother.

Partnerships with older men often brought women into contact with the conflict. Some women described the involvement of their partner in groups associated with the conflict, and importantly it was partners’ activities related to the conflict that were the reason for displacement for some participants, rather than the women’s own involvement.

*‘In the first 2 or 3 years it was good […] but then at one point he started to meet with people there, people like the [armed group] for example, and it started – he was ordered what to do, what to see […] he ended up in prison and it started – a horrible situation […]With respect to his problems, I was threatened. He is in prison, and so with respect to these problems,* his *problems, they displaced me.’ Rosa 27*

*‘I was displaced, because I was living with the first husband that I had. He was a professional soldier. And then the [armed group] entered, so there were many problems, they were using threats and putting out information that soldiers are dying, so of course I didn’t have much time together with him – and more because all the town knew that I had been with him…’—Veronica, 23*

The specific names of the armed groups have been removed from these quotes to maintain anonymity. Displacement was difficult to talk about due to associated trauma. There was also concern regarding ongoing risk in their new neighbourhoods from people associated with groups responsible for their initial displacement.

For some of the women interviewed, pregnancies took place prior to displacement, and they were displaced with their children. For others, they had become pregnant during their adolescence after their forced displacement. In the following section we will report on results of ongoing challenges in adolescent pregnancy and motherhood as a displaced person.

### Ongoing challenges in urban life: barriers to economic self-sufficiency as a means of survival—“It’s hard to raise a child at times without having anything to give him anything to eat”

The experiences of the women interviewed showed a continuous tension between the ideal of motherhood and the challenges faced in their daily survival as single adolescent mothers following displacement. On moving to the city women experienced new difficulties in the context of their motherhood now as displaced women.

*“To know that you have someone in your belly that depends on you, the little kicks, that they call you mummy, all of these are very beautiful things. […] But at the same time [its] very hard to raise a child, to raise him, at times without having anything to give him to eat. There are nice experiences of being a mum and also there are some which aren’t so nice. […] I don’t have anything for rent, I don’t have anything to eat. It’s all of these things that seem the most difficult to me at the moment”—Yessica, 21*

As this quote from Yessica shows, poverty was key to shaping experiences of motherhood. Poverty significantly constrained women’s abilities to raise their children and survive comfortably in their situation of displacement. With many displaced people arriving in Bogotá, in particular to Ciudad Bolivar where rent is cheapest, there is high competition for few jobs. Women had to become self-sufficient in providing for themselves and their children by seeking work outside of their own home, despite this not necessarily being the normal routine in life prior to displacement, where male partners would have been the economic providers. They experienced a shortage of jobs and, like many other displaced people [50] had primarily agricultural-related skills, for which there was little demand in their new environment. There were several important factors contributing towards difficulties sustaining economic self-sufficiency, which we will explore in this section.

#### (1) Barriers to education—“To go to school when you’re pregnant? Its quite complicated…”

With high competition for work, formal qualifications became vital to finding work, according to the participants narratives. Education and qualifications suddenly represented a means to improve the chances of getting a job to provide for the family. As a result, education now held a much greater importance in terms of survival than it had before displacement. This changed the implications of pregnancy during ‘school age’, where pregnancy and motherhood represent a forgone opportunity for education and therefore generate a barrier to economic self-sufficiency.

*‘[When I found out I felt] bad. Because at this age its something you don’t want. It makes everything fall apart. Your dreams, and everything. […] there were times I used to think that having a baby was ok, that you would be able to keep studying but you can’t leave your baby with anyone, […] So I felt that everything is over but I hope God wishes it is not like that. That I can keep studying.*

*‘At the moment all jobs are asking for the [qualification of finishing high school]. All jobs including sweeping the streets depend on it. So that’s how it is without studies or anything its very difficult to have a career or something. That’s how it is in this place […] still I have to fight for him [my baby]. Because of that [I felt bad when I found out about the pregnancy]. And because my dreams fall apart because of that.’ –* Nathalia, 15 who had become pregnant after displacement.

*‘To already have a baby [in the countryside] it was like something that you already know how to do. Its not like here, at least, because [my daughter] needs to study… in the city that tos and froes.’—*Mariana—36, Mother of participant

For many women, pregnancy during teenage years meant leaving education. Of all the women interviewed, none had completed high school. Although attending free-of-charge government schools, families had struggled to provide other materials necessary to attend school, such as school uniforms and books, restricting their access.

*‘I didn’t have the resources to be able to stay in school, and well, I was pregnant, it was really difficult. When I was pregnant, I had to work, and so yes, because of that I left school. […] my paternal grandparents raised me, and you know in order to study, that involves many things - uniforms, books, many things, and so yeah we didn’t have the resources to continue with school while I was pregnant.’* Veronica, 23

Furthermore, having a child meant having new financial burdens, compelling women to quit school due to the need to work and source more income.

*‘I had to see if I studied, or if I continued with my pregnancy. But yes, I decided to continue with my pregnancy and so that made me responsible for – well I had to look out for myself. So I started to work.’* Valeria, 23

The ability to attend school also depended on parental support in the form of cultural capital. This emerged as a barrier in rural settings, where education and school qualifications may be considered less important in the scheme of rural existence.

*‘I never went to school, only for a bit. I don’t know when I left for work, the people in the places where I worked helped me with learning to read and write, [..] but yeah, I never studied, I never had the opportunity to study, in my childhood no. I lived in the country with my mum, my grandparents, I didn’t have the stability to be able to study, my mother and father never told me to study.’ Rosa, 27*

Bullying from peers, actual or feared, around the time the woman discovered her pregnancy, was involved in the decision to leave school.

*‘In the school I was at yes, [there was discrimination]. I mean, there was another girl who was pregnant much earlier than me, and she left as well because [...] there was a lot of bullying, because of the fact that you’re pregnant and you’re so young, and you go to school when you’re pregnant, and I didn’t want that oh no! To go to school when you’re pregnant? Its quite complicated…’* Veronica, 23

*‘the head teacher was quite old fashioned [at my school] […] I didn’t like that he said things, like that he didn’t think pregnant women should go to school, that they should stay at home, like that - I didn’t like it. So to avoid those problems, I said to my mum that I was leaving.’* Nathalia, 15

Nathalia’s quote shows that she was afraid of judgement from her teacher, and this was her reason for leaving school. It was clear that there were barriers both to initial access to school, and in remaining in education after becoming pregnant. This restricted the ability to continue in education to secure the necessary qualifications, and subsequently put them in subordinate positions in terms of social opportunities thereafter. As a result, amongst the participants pregnancy in adolescence was considered to be more problematic once in the urban setting, giving women less of a competitive strength for accessing employment.

#### (2) Low-paid, insecure employment

There were numerous barriers to economic self-sufficiency in these women’s experiences. Labour laws limit the number of hours that can be worked under the age of 18, restricting access to the labour market (Colombian Labor Law, Art. 242). This meant that finding a source of income below this age was frequently done through alternative, sometimes irregular, means. This puts women at risk of exploitation from employers and also puts them in vulnerable or potentially dangerous circumstances to generate an income. Where there were few other opportunities for income, for some, sex-work was used as a means of earning money to support their children.

*‘I had my son at 15 in a small town where there wasn’t many opportunities. I had to do bad things, to be able to give my son what he needed.’—*Veronica, 23

*‘the only way for me to move forward was to prostitute myself, to bring in food for my son.’—*Yessica, 21

In some cases, women fell pregnant again due to engaging in sex-work for their income.

Most of the women interviewed worked in low-paid temporary jobs, with no contract. Many had to travel far to the more affluent areas of Bogota, which cost a large proportion of daily wage. With un-contracted labour, there was neither compensation for job loss, nor healthcare insurance for oneself or family.

*‘I always work really hard and earn very little. […] You work for the day and [you get] nothing else’—*Rosa, 27

The temporary, non-fixed, nature of the work available meant that women felt insecurity in their earnings and uncertainty about the future.

Another significant barrier facing the participants was that to work meant that they needed to find childcare. With limited family or social networks in a new community, this was challenging, posing another barrier to economic self-sufficiency. There was no government provision of childcare in these women’s accounts, and working took priority, where financial deprivation was the more pressing issue. Rosa, for example, a single parent, had to work late into the night, and unable to afford childcare, would leave her children alone at home. She feared that this potentially put her children at risk.

#### (3) Failures of government support systems and limited access to healthcare

Despite the establishment of programs and policies designated for the displaced population, there was little trust in the governmental aid system amongst participants.

*‘I suppose that the government say that they give help to the displaced population, that they give this, that they do that, but really it is just lies… [...] They don’t do what they say. [...] Really it’s like if you don’t work, you don’t eat, if you don’t work you don’t pay rent.’* Veronica, 23

The application process for registering displacement, in order to receive government financial support, was challenging in participant experiences.

*‘It wasn’t easy because there are things which I don’t want to say, things that they ask about the problems in your relationship, why is it like this or why you are here, it’s not easy. And there are many things they ask that you can’t answer because you don’t know or that you don’t want to say because you are frightened. It’s not easy. They threaten you, they charge you. It’s not easy.’* Rosa, 27

Rosa’s quote demonstrates the ongoing sense of fear following displacement, which presents challenges in accessing support. Enquiring into welfare schemes available to displaced people involved an appointment at a government centre, such as the Unidad de Atención de las Victimas (Unit for Attention of Victims), where staff managed cases of displacement and attempted to rectify issues with the financial aids. Trips to such centres were considered time-consuming due to the number of people applying for services. Participants felt these visits were ineffectual and meant forgoing a day of wages.

*‘You’re going to perish, waking up at 2 or 3 in the morning to get your compensation, and you arrive there for a ticket. [You wait] until around 12 or 1 in the afternoon, and you arrive and “oh look you don’t have a budget estimate, oh no” and nothing! and that’s what they do - a world of questions but nothing else. I mean that, we aren’t surviving because of the displacement [financial aids]. We are surviving because we work.’* Camila, 34—Mother of participant

The stories of the interviewees implied that these financial aids at the time had been ineffective and were not a reliable financial support.

Although all pregnant women are entitled to free antenatal services, women reported lack of information and knowledge of this service. The women interviewed were affiliated with a number of different private healthcare insurers, including the state-subsidised insurance programs. However, it emerged that there were challenges in accessing their existing registered insurance schemes due to their displacement. One participant, who was pregnant at time of interview, had no access to antenatal care for the duration of her pregnancy. Her medical insurance was registered to her home prior to displacement and she had struggled to have it transferred when she was displaced. As a result, she had not sought any antenatal medical care, having believed she was not eligible for it. Other participants also reported similar challenges in transferring their health insurance registration following displacement, affecting ability to access healthcare for them and their children.

#### (4) Lack of support from the community—gangs, discrimination and public disapproval

In the situation of displacement, people are forced to leave their community behind and with it their social support network. Women knew few people in their new neighbourhood and reported a diminished sense of community. After enduring the violence of displacement, it was difficult to trust people in the new neighbourhood who were also displaced, due to fears of association with groups involved in the conflict, or fear of being recognized, which may have had implications for safety. This lack of trust in members of the community created fear which prevented the formation of a community support network.

*‘Here people aren’t accustomed to talking to everyone, it’s like, in small towns everyone is friends with everyone, but here you see people in your neighbourhood but not everyone speaks. Really, I only know a few people […] because here the people are more mistrusting.’—*Veronica, 23

Gang activities were prominent within the new neighbourhoods according to participants, with direct encounters or general awareness of gang activities. For one participant, the father of her baby had been killed in an incident involving gang members, leaving her to raise their child alone.

Alongside general mistrust in these neighbourhoods, women described verbal abuse in the streets on account of their pregnancies.

*‘[People in the street], they mock her because she is pregnant. [...] [Pregnancy] is something that is very natural, but it's only the idea that people say it is too young.[…] It's always because of the judgement, that is why you never leave the house, that's why you keep the door shut’—*Mother of Nathalia, 15.

This public disapproval was compounded by the discrimination faced as displaced persons, due to association with the violent conflict, alongside the stigmatisation of their pregnancy.

### Limitations

Since the data presented applies only to the women interviewed living in these particular circumstances, extrapolation from these results to the general displaced population is limited and more complex. The first author is a non-native speaker which may have impacted on the interpretation of the results. This limitation was minimised through presence of a native speaking interpreter during interviews, and by relaying translations, transcriptions and analysis to the second author, a native speaker.

## Discussion

These narratives show how pregnancy and motherhood during adolescence can be contradictory. While for the women in the study adolescent pregnancy was beneficial in many ways, the positive associations of motherhood were contrasted by the difficulties encountered during and after pregnancy. The challenges facing women can be attributed to multiple clear manifestations of structural violence in the form of uneven distribution of resources and power, and their legitimisation by cultural values and practices. We will discuss how these were demonstrated in the results and how this supports the notion that structural violence and social factors contribute to adolescent pregnancy being a problem for displaced women, rather than age alone.

Pregnancy and motherhood, considered natural and beautiful, offered a way to improve existence when faced with numerous struggles of rural and displaced life. Becoming a mother could bring meaning and purpose to women’s lives, where they may not have felt their own value previously. Having children at this age hastened a perceived inevitability of motherhood and could be enabling, with gain in social status. While partnerships with men may have been transient, bringing potential for abuse, involvement in the conflict, or both, the constancy of devotion and love felt from their children was new and certain. Through this lens, becoming pregnant could be seen to represent a logical response to poverty, abuse and social insecurity, according to the gender expectations of being a woman. Pregnancy and motherhood could become a survival mechanism when faced with challenges and barbs in every direction.

Structural violence, in the forms of political violence, conflict and poverty contributes to marked resource asymmetry for rural or displaced communities, creating a social disadvantage that leads to early experiences of adversity. A cultural belief in ascribed gender roles and the belief in the beauty and inevitability of motherhood for women create the idea of pregnancy as a solution to the social issues facing these women. These beliefs in gender norms could be seen as a form of cultural violence. This system in which pregnancy is a solution therefore represents a complex play of structural and cultural violence, which adolescent women must carefully navigate.

Sexism clearly featured in narratives, in the form of power imbalances in relationships with men through financial dependency, age difference between partners, unequal decision-making power and physical abuse. Age and resource asymmetry provoked abuse, as has been described in studies elsewhere [51]. Abuse and subordination like this can be propagated by machismo cultural behaviours, with the cultural message that women are inferior to men [52]. Such practices are rooted in historical processes that have facilitated and justified differences in power and status between men and women, representing a powerful example of structural violence with cultural legitimisation [53]. Further to this, cultural idealisation of the nuclear family structure not only precipitated discrimination against single adolescent mothers but forced them out of familial households and put them into more vulnerable or dangerous positions.

Women have to forgo opportunities as a consequence of their pregnancy, including education. Limited access to education, as a result of insufficient resources, poor geographic availability, or discrimination related to their pregnancy (institutional structural or interpersonal), further restricted opportunities and the ability to provide for their own children. The ‘resource’ of education was therefore not available to them, and subsequently affected their opportunities and restricted options in terms of employment, another example of structural violence impacting displaced adolescent mothers.

Financial struggles starting in rural life pre-displacement were exacerbated by the limited access to a reliable income. Income was harder to come by due to the legal restrictions for adolescents in work and inaccessible government financial supports, which put women at risk of exploitation or compelled them to pursue potentially dangerous paths, exemplifying structural violence in limiting life choices. Another restriction to economic self-sufficiency was that those who engaged in labour outside the home were not only unpaid for their role in domestic labour but were also restricted by a lack of safe, accessible childcare. Since there were no financial gains to be made in raising one’s own children, to do so was a privilege which the households’ financial circumstances could not support. That neither provision of childcare, nor financial renumeration for domestic labour, were prioritised as a public resource demonstrates a form of discrimination of access to work for working mothers, echoing their exclusion from education, and demonstrating a further example of structural sexism.

Although healthcare was more accessible during pregnancy, uninsured status within the competition-based model of healthcare, amongst other bureaucratic and financial barriers, denied displaced adolescent mothers and their children access to healthcare following the pregnancy. This demonstrates another fundamental example of resource asymmetry within the framework of structural violence.

Adolescent mothers faced public disapproval for being perceived to be a young mother. The cultural belief that an adolescent is too young to be pregnant leads to the stigmatisation of adolescents mothers, generating abuse. Given that around 70% of the Colombian population follow the Roman Catholic faith [54] alongside religious and social conservatism among certain sectors of the population [55], it could be considered that the public might view adolescent mothers as morally deviant, as the pregnancy demonstrates that they had engaged in sex while unmarried. Such belief systems could conclude that women who become pregnant at school age warrant punishment and these systems may sustain discrimination of younger mothers, both within institutions and interpersonally. Members of a community may believe that women who are pregnant at school age set a bad example for other students, perpetuating the commonly-held belief that these women should not be able to remain in school following the discovery of their pregnancy [56]. This process acts to justify the structural violence accounting for their denial of education and other public resources. The denial of education for adolescent mothers and their subsequent exclusion from the professional job market, ensures the perpetuation of the image of adolescent mothers as being ‘unsuccessful’ by conventional standards of their society. This could be considered a case of structural violence that helps maintain certain notions of cultural violence, in what is an example of a self-fulfilling structure explained by Galtung [40].

Another important finding was that displacement from rural to urban settings was associated with a transformation of mentality. Experiences of urban life after displacement seemed to reveal to participants the other potential opportunities that might have been possible to them in urban settings, reshaping their expectations from life. This included a perceived improved access to education and different possibilities of employment compared to rural life prior to displacement. Moreover, competition for work in the urban setting meant that education was more important for survival and existence, and offered the chance to change their circumstances for the future. This affected perceptions of pregnancy in adolescence, since pregnancy at school age was incompatible with completing school and therefore it presented more challenges post-displacement than in rural settings. This indicates that some of the problems facing these women stem from the existing class structures within Colombian society, alongside the issues generated by their displacement and resettlement. Coming from a rural setting in which public services are neglected, as well as being impoverished, working class and female, put these women at a social disadvantage even before their displacement. This can have implications in both their becoming pregnant, the consequences of that pregnancy and also the ability to manage parenthood thereafter.

Pregnancy and motherhood during adolescence can be understood to represent both a safety net and a trap in the context of the lives of displaced women due to the multitude of structural factors that they encounter in daily survival. It could be argued that if these structural factors were improved, then adolescent pregnancy should not have to be a problem for those who follow this route.

These structures require careful and considered approaches to address. Global institutional framing of adolescent pregnancy as an ‘infringement of Human Rights’, as described by the UNFP, could be seen to fail to acknowledge this complexity and the positive elements that pregnancy and motherhood can bring to women in such contexts. In fact, by perpetuating a negative view and victimhood of adolescent mothers, the continued problematisation of adolescent pregnancy may itself curb opportunities for adolescent mothers as another example of structural violence, enacted on a much larger scale. This, in itself, could infringe on women’s existence and reproductive choices.

Importantly, policy makers must consider that while social disadvantage persists, pregnancy during adolescence will continue to be viewed as an attractive option and therefore measures aimed at preventing pregnancy will not offer a whole solution. Policy related to adolescent pregnancy for the displaced population should focus on improving life before and after adolescent pregnancy for displaced women. Priorities should include improving access to healthcare, education and public services such as childcare, as well as streamlining the process for accessing the financial assistance programmes, in order to support financial self-sufficiency and break the cycle of intergenerational poverty. Further to this, work on challenging gender stereotypes, and addressing the machismo culture throughout the community is an essential part of addressing the challenges faced by this group.

## Conclusion

This work illustrates how structural violence features strongly in the lives of adolescent mothers throughout the narratives of early life, pregnancy and daily survival as mothers in displacement. This upholds that problems surrounding adolescent pregnancy and motherhood result from social structures. However, the representation of adolescent pregnancy as a problem by global and national institutions can reinforce cultural and structural violence, potentially causing more challenges for adolescent mothers whilst also undermining the positive experiences that pregnancy can bring in such situations.

The unequal opportunities brought about by mass institutional and interpersonal discrimination, as well as the culturally perpetuated prejudices towards young mothers, undoubtedly contribute to the challenges of motherhood during adolescence. These factors can be understood to cause avoidable suffering, which these women must continually endure, both before, during and after the direct violence of their displacement.

In light of these findings, the notion that age itself is problematic in adolescent pregnancy should be reconsidered. Instead, more attention should be given to the systematic disadvantages and inequalities brought about by culturally legitimised structures that negatively affect women who become pregnant during adolescence. Existing policies regarding access to education, healthcare and government relief programmes are not working for adolescent women who are pregnant or mothers in displacement. As peacebuilding efforts progress, attention should be given to address the structural issues underlying the challenges facing rural and displaced adolescent mothers. Importantly, policymakers must consider the complexity of the life circumstances of displaced adolescent mothers, rather than using blanket policies that can perpetuate discrimination and contribute to further difficulties for this population group.

## Data Availability

The datasets used and analysed during the current study are available from the corresponding author on reasonable request.
